# Evaluation of online interprofessional simulation workshops for obstetric and neonatal emergencies

**DOI:** 10.5116/ijme.6342.9214

**Published:** 2022-10-31

**Authors:** Namrata Prasad, Shavi Fernando, Sue Willey, Kym Davey, Jennifer Hocking, Atul Malhotra, Arunaz Kumar

**Affiliations:** 1 Department of Obstetrics and Gynaecology, Monash University, Melbourne, Australia; 2 Monash Nursing and Midwifery, Monash University, Melbourne, Australia; 3 School of Nursing, Midwifery and Paramedicine, Australian Catholic University, Melbourne, Australia; 4 Department of Paediatrics, Monash University, Melbourne, Australia

**Keywords:** Interprofessional education, simulation-based education, online learning, learning through modelling, obstetric

## Abstract

**Objectives:**

To explore student perceptions of learning and interprofessional aspects of obstetric and neonatal emergencies through online simulation-based workshops.

**Methods:**

This qualitative study was conducted at Monash University, Australia. Data were obtained from six separate online Obstetric Neonatal Emergency Simulation workshops held between May 2020 and August 2021. A total of 385 students attended and were invited to participate in the study by completing an online survey two-three weeks later. Of the attendees, 144 students completed the survey (95 medical, 45 midwifery), equating to a response rate of 37%. Survey responses were downloaded from online survey platform and separated into medical and midwifery responses. Thematic analysis of data was performed using a coding framework, resulting in development of themes and subthemes.

**Results:**

Main themes were adaptability, connectivism, preparedness for practice, experiential learning, learning through modelling and dynamics of online interaction. Students reported that online workshop was a useful alternative method to experience simulation-based learning, increase their readiness for clinical practice and foster positive interprofessional relationships. Consistent with existing literature evaluating similar in-person programs, midwifery students were most interested in interprofessional interaction (predominant theme: dynamics of online interaction), whilst medical students were more concerned with developing clinical skills (predominant themes: learning through modelling, experiential learning).

**Conclusions:**

Online learning may be a useful and convenient way of delivering interprofessional simulation-based education during the pandemic, in remote areas and as an adjunct to in-person teaching. Future studies should evaluate the impact of online learning with a mixed methods study and in comparison, to in-person programs.

## Introduction

The development of clinical skills during training years is essential in establishing a competent, safe and efficient healthcare workforce.^1^ In obstetrics and gynaecology, clinical skills education has been demonstrated to improve patient outcomes and satisfaction.^2^ Simulation-based education (SBE) and interprofessional education (IPE) have emerged as effective methods of team-based clinical learning.^2^

In the context of obstetric emergencies, combining IPE and SBE is ideal due to the involvement of a multidisciplinary team in their management and the high-risk nature and rarity of such situations in real life. A program that incorporates aspects of both IPE and SBE is the Obstetric and Neonatal Emergency Simulation (ONE-Sim) workshop.^3^^,^^4^ This workshop simulates obstetric and neonatal emergencies, aiming to improve situational awareness, preparedness for practice, interprofessional communication and teamwork between medical and midwifery disciplines.

The novel coronavirus (COVID-19) pandemic resulted in a shift in all forms of interactions, including in social^5^ and educational contexts. Throughout the pandemic, there has been increased uptake of social networking and video conferencing platforms in order to maintain a sense of connectedness.^5^ Across universities, video conferencing has been used as an alternative platform to deliver teaching that has traditionally been in-person, such as small group sessions and tutorials. At our university, the ONE-Sim workshops were also transitioned to an online format, and we published our initial experience using this format in 2020.^4^

In light of the ongoing COVID pandemic, there is a growing body of literature evaluating SBE delivered in an e-learning format.^6^^-^^8^ These studies demonstrate online SBE is able to achieve high levels of student satisfaction, concept understanding and psychomotor skill development whilst acknowledging limitations of online simulation, such as technical feasibility, particularly in low and middle-income countries where technological resources are potentially fewer, and logistical issues.^7^ Similarly, since the onset of the pandemic, multiple studies have evaluated online IPE programs in healthcare. These studies demonstrate IPE delivered in online settings is able to help foster positive attitudes towards teamwork, interprofessional communication and collaboration. Students also felt that learning through online IPE could improve outcomes for their patients.^9^^-^^11^

With ongoing recommendations for social distancing where possible in many countries, the need for effective online teaching alternatives remains. Although in-person SBE has long formed an integral part of obstetric training and education and has been heavily appraised in the literature,^12^ there is a scarcity of studies evaluating online SBE in obstetrics. Moreover, although there is increasing evidence supporting the use of online IPE and SBE programs individually, there are very few studies evaluating a combined online IPE and SBE program. Evaluation of such programs is particularly important in the field of obstetrics, where combining IPE and SBE is ideal due to the multidisciplinary, high-stakes nature of the specialty. Finally, whilst the existing literature evaluates learning outcomes from online SBE or IPE programs, there is a limited exploration of the influence of the online setting on pedagogies.

Our study aims to address this gap in the literature by evaluating an online combined SBE and IPE program specific to obstetric training from the perspective of medical and midwifery students to answer the following research questions:

1.   How do students perceive learning in the novel online format?

2.   What are students’ perceptions of obstetric and neonatal emergencies when observed through online simulation?

3.   How do medical and midwifery students perceive the interprofessional aspects of management of these emergencies when delivered online?

## Methods

### Study design and participants

An inductive qualitative research design based on post-workshop survey data was employed. The study population consisted of all fourth-year medical students at Monash University on their Obstetrics and Gynaecology rotation (n=250 per year), and midwifery students from Monash University (n=49 per year) and Australian Catholic University (n=60, only invited to 2021 workshops). These students were invited by email to attend one of the six online ONE-Sim workshops held between May 2020 and August 2021 and to subsequently complete a post-workshop survey. A total of 385 students (269 medical, 116 midwifery) attended one of the six online ONE-Sim workshops between May 2020- August 2021.

Given the study was based on a novel initiative where students participated in online obstetric and neonatal simulation, the study participants’ experiences would be highly specific. The sample, therefore, consists of participants with dense specificity, enhancing the information power of the data.

Students were included in our study if they attended one of the online ONE-Sim workshops, consented to study participation and completed the post-workshop survey. Of the 385 students who had attended one of the six online workshops, 144 students (95 medical, 49 midwifery) participated in the study, equating to a response rate of 37%. The low response rate is likely attributable to the delay between students attending the workshop and the post-workshop survey being sent to students. We had designed the study such that the survey would be sent 2-3 weeks after attending the workshop, so that students had the opportunity to reflect on their learning from the workshop and the interprofessional interactions they observed. However, as a result, students’ motivation to complete an optional survey a few weeks after attending the workshop may have been reduced.

Low risk ethics approval for this study was obtained from the Monash University Human Research Ethics Committee. A consent form and explanatory statement detailing study information were attached to the email inviting the study population to attend the online workshop. The consent form clearly documented that study participants were free to withdraw consent at any time during the project without affecting their academic progression or assessments.

### Survey

The survey questions are listed in the Appendix. The survey consisted of four open-ended “free text” questions aimed to address the study objectives and questions relating to demographic information such as age, gender and professional group (medical or midwifery). The surveys were drafted by the medical and midwifery educators on the research team and later reviewed by faculty members and students (who did not participate in the study) for validation. The process involved multiple rounds of review and was carried over a duration of 3 months. Free text questions were chosen to get thick descriptions of how students perceived learning by observation of simulation scenarios. To evaluate students’ perceptions of learning in an online format, we developed a question exploring how learning via video conferencing will influence students’ future clinical practice. To understand how students perceive obstetric and neonatal emergencies when observed online, we developed questions comparing students’ experience of in-person obstetrics teaching with the online workshop. To explore students’ perceptions of the interprofessional aspects of obstetric and neonatal emergencies in an online setting, we developed questions pertaining to their experiences of interacting with other professional groups online and whether they would prefer intra-disciplinary to interprofessional teaching when delivered online. In the study, both the research and survey questions were quite specific. Hence, it was anticipated that there would be sufficient information power to provide rigor to the study.

### Data collection

Across six independent workshops held between May 2020 and August 2021, a total of 95 medical and 49 midwifery students participated in the study (n = 144). Study participants completed an online survey via “Qualtrics” (Qualtrics, Provo, UT), sent to their student email IDs by the ONE-Sim administrative staff 2-3 weeks after completing the online ONE-Sim workshop. The survey was sent at this time point so that students had the opportunity to reflect on their learning from the workshop and the interprofessional interactions they observed. Students were informed that all responses would be anonymous. NP was given access to the survey responses on Qualtrics, and downloaded and collated responses into an Excel spreadsheet verbatim. Since participants were made aware that survey responses were anonymous and had no effect on their academic assessment, they would likely feel more comfortable expressing their true thoughts and feelings in the survey responses. In combination with relatively specific survey questions, the quality of dialogue is strong, ensuring sufficient information power. Credibility was ensured through data and investigator triangulation. Data was triangulated by using survey responses relating to six separate workshops held at different time points between May 2020 and August 2021. Investigator triangulation was achieved through two different investigators independently performing analysis of the data and multiple different researchers reviewing the final themes yielded from thematic analysis.

### Study setting

**Figure 1:**
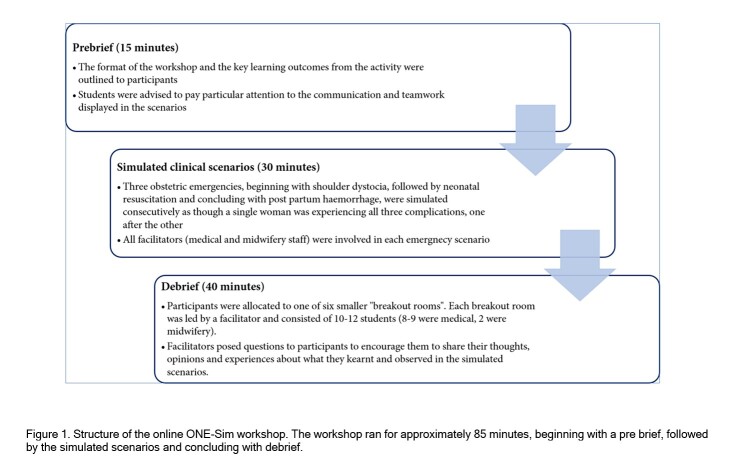
Structure of the online ONE-Sim workshop. The workshop ran for approximately 85 minutes, beginning with a prebrief, followed by the simulated scenarios and concluding with debrief.

The study was performed at Monash University, Australia. The online ONE-Sim program has been described in detail previously.^4^ In brief, the workshop was conducted online via a video conferencing platform, “Zoom”. The workshop was approximately 85 minutes in duration. The structure of the workshop is outlined in Figure 1 and includes a pre-brief followed by simulation scenarios and ending with a de-brief.

At the time of attending the online ONE-Sim, both medical and midwifery students were undergoing clinical placements in the birth suites of various affiliated hospitals. However, due to differences in course length and structure, medical students had completed less than 12 weeks of clinical placement, whereas midwifery students had a longer stint in clinical placements. Due to pandemic restrictions, both groups had received limited structured or large-group in-person learning in the preceding months.

### Analysis methods

Within the Excel spreadsheet, data were separated into medical and midwifery student responses. Thematic analysis was performed on each set of survey responses. A coding framework was first created.^13^ NP and AK inductively and independently coded the survey responses. The qualitative dataset was found to be comprehensive enough to identify recurring codes and patterns to generate adequate information power. A set of themes was independently developed from these codes. AK and NP then agreed upon these themes, closely reflecting participants’ own responses. The second phase of the analysis involved identifying subthemes nested within each higher-order theme. The third phase involved negotiation, discussion and collective review of data by NP, AK and other co-investigators JH, SW, KD, SF and AM to yield final themes.

## Results

### Thematic analysis

Major themes and sub-themes are described below in the order of what was considered most dominant by both groups (Table 1), the medical group (Table 2) and the midwifery group (Table 3). Common themes between the groups related to “adaptability”, “connectivism” and “preparedness for practice”. The predominant themes identified in medical student evaluations were “experiential learning” and “learning through modelling”. The major theme predominantly identified in midwifery students was “dynamics of online interaction”.

### Adaptability

The COVID-19 pandemic and the requirement to socially distance represent circumstances outside the student's control. Adaptability is related to students’ capacity to cope with a lack of in-person teaching and learning through an alternate delivery of teaching, e-learning, with which they may be less familiar.  The subthemes within this main theme were “comfort with e-learning”, “responding to dynamic circumstances”, and “future applications of e-learning”; e.g., “I thought given we couldn’t be there in person, nor practice our potential future role in the scenario, the Zoom session was an excellent alternative.”; “I think that this was beneficial …as we have done a lot of simulation …so this was a good opportunity to consolidate our knowledge in a different way, through reflection.” (MED 12, Table 1).

### Connectivism

Connectivism is the concept of digital technologies, such as the video conferencing platform employed in this study, providing new avenues for learning. Specifically, learning exists as a “network” of learners (in this study, midwifery and medical students) who are influenced by technology and social interactions. The subthemes within this main theme are “facilitators as nodes in the learning network” and “diversity of opinions”; e.g., “Through interacting with different professions and educators, I feel that it was an eye-opening experience.” (MW 4, Table 1); “I found it eye-opening to have a workshop alongside the midwifery students. They have a lot of input and suggestions when it came to the content. I got to ‘see’ what are the thoughts in their minds during an emergency situation.” (MED 34, Table 1).

### Preparedness for practice

“Preparedness for practice” revolves around the simulation’s ability to increase student confidence in approaching clinical situations in work-based placement. The theme explored greater awareness of their own role in a clinical setting, both as a student currently and their projected role as a qualified practitioner, as a result of the simulation. Additionally, it encompassed the interprofessional rapport and relationships formed through exposure to and interaction with students from other disciplines as part of the program.  The subthemes within this main theme were “role self-awareness within a team, both as a student and in their projected role” and “fostering positive working relationships at a grassroots level that will be reflected in future practice”; e.g., “…fostering mutual respect at a student level is a real positive” (MW 18, Table 1); “I think I have better insight into how to navigate my role in an emergency, which will definitely be important in my future practice” (MED 89, Table 1).

### Experiential learning

Experiential learning refers to the real-time, hands-on aspects of simulation-based education and the fidelity of the simulation to real-world scenarios. The main theme of experiential learning was divided into further subthemes relating to the “personal stress response to real-time emergencies” associated with enacting a simulated emergency oneself, as well as “learning by doing”, where students are referring to the practical experience of developing core skills through the workshop.  Additional subthemes within this main theme were, “lack of physical interaction”, “clinical skills confidence” and “creating realism through e-learning”; e.g., “It was a bit difficult to learn this over zoom, especially because we didn’t have the opportunity to be hands on” (MED 1, Table 2); “…that lack of adrenaline means a valuable facet was lost from the demonstration” (MED 32, Table 2).

**Table 1:** Themes and subthemes common to both medical and midwifery students

Medical Students	Midwifery students
**1. Adaptability **	
· **Comfort with e-learning** *MED 2: “I think it will make me more willing to participate in Zoom conferences in the future because I know how useful they can be and how well they can be delivered.”* *MED 11: “Will become more used to working on a technological front.”* *MED 67: “Zoom, I think, will inevitably be a much larger part of our jobs well into the future, not just while the pandemic is still rampant. As such, it is a good skill to learn how to interact with your peers and other professions via this method.”*	· **Comfort with e-learning** *MW 24: “I think after studying online most of us are becoming used to learning this way”*
· **Responding to dynamic circumstances** *MED 12: “I thought given we couldn’t be there in person, nor practice our potential future role in the scenario, the Zoom session was an excellent alternative.” * *MED 28: “I guess doing a clinical skills workshop online last year would’ve been thought to be impossible and yet it’s worked…” MED 7: “Not as good as in person but made the best of a difficult situation!”* *MED 84: “While there's something lost in being unable to experience a run-through physically… one benefit of being on Zoom. is that we can see a smooth proceeding from one emergency to another, with the focus on each situation well-highlighted”*	· **Responding to dynamic circumstances** *MW 15: “It helps to be more flexible and innovative in your approach” MW 11: “Of course it is probably more beneficial over all to have in-person interactions but the Zoom conferencing did allow a very accessible experience” MW 4: “I would have preferred to have an in-person session however, despite the difficulties, the teaching team did get through adequate information”* *MW 7: “I think the session was good given the circumstances…”* *MW 22: “While face-to-face learning where people get to actively participate will always be the best option, when that is not available, this is not a bad second choice in my opinion.”* *MW 25: “importance of teamwork and communication in emergency situations still as effective over zoom!”* *MW 26: “I don’t think it *[Zoom format] *really affected as much-was nice to be able to do it from the comfort of my own home.”* *MW 43: “I still believe that in person interprofessional workshops… are more beneficial in terms of … practical skills. However, given the current climate, zoom video conferencing is a great substitute. Zoom calls are also easier to attend and therefore increases students ability to join the workshops.”*
· **Future applications of e-learning ** *MED 2: “…I think it will especially help me engage with rural and remote patients and be very willing to do this.”* *MED 30: “Even when there is no pandemic, most of us does not get equal clinical exposure…having these clinical teaching sessions via Zoom…helps the students who potentially would have missed out.” MED 31: “…this is something that will continue post COVID, especially with the funding the government has put into telehealth…” * *MED 46: “It was convenient and the perspective from watching through a video was good, and probably better than with multiple other students.”* *MED 47: “I think it would be really good in the future to have the video provided prior to an in person session so that students can gain some knowledge in order to participate fully in the in person session”*	**Future applications of e-learning** *MW 19: “I actually liked the online format…I think I would be more likely to go when it’s online”* *MW 19: “I think interprofessional workshops are super valuable and zoom is a good way to make them convenient”* *MW 34: “I think that this was beneficial to me personally as we have done a lot of simulation of these situations in our units last semester and so this was a good opportunity to consolidate our knowledge in a different way, through reflection.”*
**2. Connectivism **	
· **Facilitators as nodes in the learning network ** *MED 2: “I think having several ‘experts’ on a Zoom talking from different perspectives …is really valuable”* MED 30: “…it is important to have a live session with a midwife or a doctor to debrief the video and go through questions.” MED 15: “…we could clarify our questions during the debrief with Dr *** very easily…”	· **Facilitators as nodes in the learning network ***MW 4: “Through interacting with different professions and educators, I feel that it was an eye-opening experience.” MW 15: “It was great learning from experienced practitioners…”* *MW 42: “…the communication and teamwork discussion…was great from the lecturers”*
· **Diversity of opinions** *MED 34: “I found it eye-opening to have a workshop alongside the midwifery students. They have a lot of input and suggestions when it came to the content. I got to ‘see’ what are the thoughts in their minds during an emergency situation.” * *MED 11: “I enjoy working with other professions such as midwifery students as it gives a more holistic view and can hear their opinions”* *MED 15: “…doing the debrief on Zoom is possibly more systematic than a live setting as we are all applying ourselves in one call and able to ask questions…” * *MED 19: “The experience from various professions allows a broad approach to learning and should be encouraged.”* *MED 18: *“*I think the set up of the Zoom, with break out groups was very good, it allowed us to debrief what we had encountered…”* *MED 29: “It was useful to hear insights from midwives/midwifery students during group discussions.”* *MED 13: “Fantastic to learn about what midwives do. We should do it with more professionals.”* *MED 47: “It was really good to hear from midwifery students as they can provide different expertise from what we can”* *MED 96: “I think it is beneficial to have students from different professions as we all have different strengths and weaknesses.”* *MED 95: “as a medical student I really look forward to interdisciplinary tutorials/workshops because I really like getting to know students form other disciplines!! We're curious about what their role is in the clinical setting, what their training is like, where they're placed…”*	· **Diversity of opinions ***MW 1: “The students can ask questions more confidently and get answers by sharing their knowledge.” MW 5: “…after attending this Zoom, I have learnt a lot about what medical students think during an emergency and what their scope of practice is.” MW 18: “I love the opportunity to learn and get a small glimpse at the medical students learning/thoughts/experiences.” MW 11: “…I did find it very interesting to hear how med students’ views on procedures and policies differed and found that very helpful in itself…” MW 17: “I REALLY enjoyed working with medical students, I think it was very interesting to understand their point of view. This should be done more in the future.” MW 11: “It was also great to hear other perspectives of labour and birth management” MW 18: “The more opportunities I have to learn (and from different perspectives) the better midwife I will become. MW 46: “It was good to hear about the scenario shown form the prospective of med students as well as midwifery students as we see these situations differently based on our own scope of practise, as well as having different experience levels” MW 43: “…the zoom conferencing was a great way to share opinions and information”* *MW 31: “My group had great discussions regarding the pros and cons of the scenario and how it can be done better.”* *MW 31: “I feel that it is good to have a mixture of both midwifery and medical students to allow for us to learn from each other and learn different ways of managing these situations.”* *MW40: “I enjoy having the interprofessional workshops, provides an opportunity for midwifery and medical students to understand the role of each other more clearly.”*
**3. Preparedness for practice **	
· **Role self-awareness within a team, both as a student and in their projected role ** *MED 9: “Good to have things explained before getting to the ward and being in the middle of it not understanding what’s going on or how you can help”* *MED 14: *“*For medical students we’d at least have an idea of what to expect and potential roles we can have…” MED 14: “Considering we have not gotten any in person experience yet, this zoom teaching is very helpful …and prepare us for attending one either end of this sem or next sem”* *MED 89: “I think I have better insight into how to navigate my role in an emergency, which will definitely be important in my future practice”* *MED 49: “*[learnt] *The role students can play if they are in an emergency situation. The role doesn't have to be outside their scope of knowledge but could include informing the mother or support person of what is going on…”*	· **Role self-awareness within a team, both as a student and in their projected role** *MW 22: “As a student I will ensure I am identifiable to the other professionals I may be working with and be prepared to play a role during an emergency situation.”* *MW 48: “*[gained from the session] *more self-awareness in my practice”*
· **Fostering positive working relationships at a grassroots level that will be reflected in future practice ** *MED 30: “It also helps establish professional relationships and have a sense of team early on with the midwifery students.” * *MED 24: “…it would definitely be better to have the opportunity to work with those from other professions as well, as we can learn more about their roles which then facilitates teamwork in the future.”* *MED 83: “As we will be working closely together on placement and later in our careers it is nice to build that relationship and respect early.”* *MED 49: “I think it is good practice to begin interconnected learning between disciplines early on”* *MED 68:” I really like the fact that we have interprofessional interactions…it's so important for us to realize each others strengths for when we will work together later on once we graduate.”*	· **Fostering positive working relationships at a grassroots level that will be reflected in future practice ***MW 8: “I think workshops like these help start to build that teamwork at a student level, which I think benefits our future professional relationship and perceptions of each other…” MW 16: “I think I will be more open to merged practice settings.” MW 18: “…fostering mutual respect at a student level is a real positive”* *MW 16: “Given the need for our professions to work in partnership for each woman, I think this style of integrated classroom learning activities are hugely beneficial for both areas of study!”* *MW 28: “I think it is much more beneficial to run this with both professions. We need to work together in the next couple of years so why not start at university.”*

MED: Medical student, e.g., MED 58 indicates a survey response from the 58th medical student who completed the survey; MW: Midwifery student, e.g., MW 24 indicates a survey response from the 24th midwifery student who completed the survey

**Table 2:** Themes and subthemes predominant amongst medical students

Medical Students	Midwifery Students
**4. Experiential learning**	
· **Personal stress response to real-time emergencies ** *MED 32: “…that lack of adrenaline means a valuable facet was lost from the demonstration”* *MED 53: “When in person … It would be good to be challenged to think what the next step would be after each thing is done.”*	· **Personal stress response to real-time emergencies** *MW 18: “In person is great to …get a feel for a true emergency situation”*
· **Lack of physical interaction ** *MED 10: “…there is something invaluable about interacting with strangers in a shared space rather than a virtual one, which can feel a bit impersonal” * *MED 9: “Zoom doesn’t allow for those less formal conversations that may happen in person between disciplines which often allow you to bond…”* *MED 82: “I don't really like it *[online interprofessional interaction]* as much as it feels a lot more formal and awkward than in person”* *MED 83: “…it is always nice to meet face to face with other students to build that relationship and rapport.”* *MED 71: “It feels more awkward to participate on zoom and communication can sometimes be hampered.”*	· **Lack of physical interaction ***MW 9: “I think that it is difficult to build interactions and practice simulations for ourselves to practice interprofessional relationships and working as a team”MW 9: “The workshop was good to talk to medical students…however I feel this would have been better in a face-to-face interaction” MW 8: “I still think I prefer the in-person interactions over the zoom.”*
· **Learning by doing** *MED 1: “It was a bit difficult to learn this over zoom, especially because we didn’t have the opportunity to be hands on and try to complete these emergencies ourselves.”* *MED 3: “…how its management occurs in real life. Learning this via zoom was not very effective in general as I feel watching someone perform tasks is much less helpful than getting to practice in a simulated environment.” * *MED 16: “I think in person where I can get involved would be extremely valuable…” MED 17: “Being in person probably allows us to be a bit more hands on…” * *MED 42: “…I do think it would be better in person as you can get a greater understanding with hands on experience.”* *MED 8: “…we were not able to be hands on and learn by doing.”* *MED 53: “When in person…we can take turns to actually do what was done in the video on models (shoulder dystocia manoeuvres, resus etc).”* *MED 84: “…there’s something lost in being unable to experience a run-through physically (so we can develop a sort of muscle memory…”*	· **Learning by doing ***MW 3: “Watching via Zoom is obviously nothing like practising the skill itself, so it was difficult to increase my confidence in the recognition and management of these emergencies by Zoom observation only” MW 7: “…would have been a lot more beneficial had we done the sessions in person as we would have been able to do hands on practice (which I learn better doing compared to watching others do the simulation).” MW 18: “…in person running through the steps physically provided greater learning for me…”MW 7: *“*…for me personally I am much less likely to remember what everyone does in different scenarios if I can’t actually be a part of the physical simulation .”*
· **Clinical skills confidence ** *MED 36: “Learnt how to perform sensitive examinations such as bi manual, speculum and vaginal exams…”* *MED 28: “I learnt examination technique and what to look for and skills that will definitely help me feel more confident with approaching the clinical environment…”*	· **Clinical skills confidence ***MW 14: “Developed a better understanding of VE technique and use of speculum”MW 16: “Variations in vaginal examinations, tips & tricks on how to best perform these examinations with comfort, ease and accuracy for the woman.”MW 13: “I learnt how to do a vaginal exam, high & low & dilation of the woman. Along with the delivery of the baby and the placenta.”*
· **Creating realism through e-learning ** *MED 25: “The simulation was very realistic and was able to convey the emotion, intensity…in such a situation”* *MED 24: *“*It was also good to see the different emergencies play out at the same time, and it gives us a good perspective on how an obstetric emergency can play out in real life.”* *“…it was… an opportunity to feel like you were right in with the team…”* *MED 22: “…the video we were given to watch was well done and quite realistic.”* *MED 73: “This was my first insight into more real time management of an obstetric emergency…I thought the video was well filmed.”*	· **Creating realism through e-learning *** MW 7: “…it was good to see everything happening as though one scenario as opposed to three different situations”*
**5. Learning through modelling **	
· **Interpersonal interactions** *MED 12: “I learnt how a well-drilled team makes management of an emergency flow smoothly…everyone in the group was aware of not only their own skills, but the skills of all team members, and this allowed leadership to flow to the right person for the right task”* *MED 5: “I was able to see the interventions that I have studied play out in a well-oiled team environment…” MED 33: “I was able to view the procedure from a ‘fly on the wall’ perspective and was able to focus on what was happening everywhere…rather than…if I was participating in the emergency simulation myself”* *MED 42: “I thought the ONE-Sim video that we watched was an extremely valuable learning resource, as it gives us an ability to visualize how an obstetric emergency actually takes place and we get to see how the most experienced professionals do it.” * *MED 14: “From what we’ve seen, there’s good rapport between the health professionals present and during the process there’s good communication to ensure efficiency with management.”* *MED 18: “It was good at showing us how midwives, obstetricians, junior trainees and paediatricians all work together and have designated jobs and how they communicate most effectively!”* *MED 25: “…does enable us to see good examples which we can mimic at a later opportunity.” * *MED 64: “I have learnt about… the manner in which people work and communicate effectively…learning via Zoom worked well for this workshop, as it allowed to us learn and observe without having to completely focus on doing an activity which may have otherwise resulted in us giving less focus to the content.”*	· **Perceiving individual’s projected roles ***MW 18: “…these sessions have been a fantastic way to start to think more in depth about my role and how to act/respond in these situations” *
· **Intrapersonal emotional regulation** *MED 39: “…the environment in an obstetric emergency can rapidly degenerate into one of panic…we need to be extremely proficient in our approach to an obstetric emergency so we can collect ourselves and respond adequately”*	· **Interpersonal interactions ***MW 9: “I think this will improve my communication skills such as clinical handovers as you can see the importance of giving succinct information in obstetric emergencies”MW 6: “It was useful…as it reminding me that there are multiple professionals involved in a situation and everyone should work together to fulfill their tasks and improve the outcome for the patient” MW 5: “By attending the zoom, I have had an opportunity to observe the communication and roles among different health professionals, which could be quite hard when attending in person”*
· **Patient centredness ** *MED 34: “I learnt about the importance of communicating with the mother about what is occurring in an emergency”*	· **Intrapersonal emotional regulation and demeanour ***MW 5: “I think I’ll be more calm during emergencies. By viewing the management from a third person’s angle, I can see … what are occurring at the same time, which means that if I’m the leader, I can make sure these things are done in the same situation*

MED: Medical student, e.g., MED 58 indicates a survey response from the 58th medical student who completed the survey; MW: Midwifery student, e.g., MW 24 indicates a survey response from the 24th midwifery student who completed the survey

**Table 3:** Theme and subthemes predominant amongst midwifery students

Medical Students	Midwifery Students
**6. Dynamics of online interaction**	
· **Dominance of a professional group** *MED 39: “I just do not think it was very conducive to have the midwifery students interrupting the workshop constantly…the midwifery students in my breakout session were constantly harping on the 1 or 2 points the entirety of the discussion session” MED 39: “…a whole bunch of eager midwifery student barrage the chat function and constantly undermining the consultants due to mismatch in what they were apparently taught. It would be hard to tell if those students would have been equally disruptive in an in person session instead.” * *MED 69: “…unfortunately our session… was filled with midwife students just slandering medical students. Multiple comments saying that medical students are not women centric like midwife students, and additional comments about our inability…Unfortunately the only environment was much more toxic and just made me feel very inadequate, unsupported, judged, and very uninspired about interprofessional learning.”* *MED 70: *	· **Power imbalance relating to their greater knowledge and clinical experience than the medical student *** MW 3: “I think it was difficult being in a group of medical students, as I have accouchered many births as a final year midwifery student…however for many of the medical students, they have never even seen a birth…they struggled to critically evaluate the scenario… medical students learning this content for the first time and the midwifery students wanting to fine tune…”* *MW 35: “it was interesting from a novice midwife perspective to understand the limited level of obstetric knowledge medical students may have prior to placement/clinicals in the birth environment. I also believe it would be a great learning opportunity for them to understand just how much pre- registration training we have in neonatal emergencies, and that much of the management can and is midwife initiated in real life.”* *MW 28: “I also felt that a lot of the midwifery students answer most of the questions as we are confident with the theory but I am not sure the medical students were given enough opportunity to discuss.”* *MW 37: “I really liked that the registered Doctors told the medical students that they can learn a lot from midwives and going forward I look forward to working in a multidisciplinary team where there is mutual respect.”* *MW 35: “I questioned the approach of the OB during the workshop and whether optimal care was being provided and will take that with me to try to ensure that as midwives, we ensure we enhance that objective through advocation.”*
· **Equality of opinions** *MED 22: “…perceived imbalance. There were only 2-3 midwifery students and I barely heard from them during the session” *	· **Equality of opinions ***MW 13: “…the debriefing proved less interaction with medical students. I found it disappointing their lack of involvement. Medical students need to understand as midwives they are there to work with and need their opinions for reflective …learning”* *MW 14: “…would have liked more input from medical students to hear from their experiences and thoughts” MW 8: “I think the main takeaway is that both midwives and doctors can … value each other’s opinions and experiences.”* *MW 22: “…many students seemed shy or hesitant to participate…it would have been interesting to learn more about the medical students perspective in this situation” * *MW 42: “I would of loved to have heard more from the medical students”* *MW 42: “There would be a greater discussion I think if it was just midwifery or medical student run, but the idea of it being together makes sense.”* *MW 47: “…I did find that conversations amongst students were more midwife led than medical student led.” *
· **Contribution from more clinically experienced midwifery students leading to increased learning by medical fraternity ** *MED 37: “I think it was good to gain insight and knowledge from midwifery students, they clearly know much more than us medical students…”* *MED 3: “I think the presence of the midwifery students helped and gave us a different perspective…as medical students, our knowledge was much poorer than midwifery students…” MED 38: “The midwifery students have a lot more knowledge and experience in this aspect so not sure how a course with only med students might run” * *MED 72: “As a med student it was really helpful to learn from the midwifery students who have had more a lot more experience in this area”* *MED 68: “There is so much to learn…especially because midwifery students know so much more about obstetrics and us medical students are only really on placement for 8 weeks so we don't have as much exposure and experience as them”*	· **Awareness of clinical challenges ** *MW 18: “I would hope that interprofessional interactions are respectful and an opportunity to share and learn from each other-in placement my experience is that this is not always the case”* *MW 36: “I think that it was a great idea and helps to break down the hierachy between midwives and doctors”* *MW 43: “I think that working with different future health care professionals is important. It is rare to have that opportunity and I believe that is what contributes to the divide between professionals and the clinical hierarchy that often exists.”*

MED: Medical student, e.g., MED 58 indicates a survey response from the 58th medical student who completed the survey; MW: Midwifery student, e.g., MW 24 indicates a survey response from the 24th midwifery student who completed the survey

### Learning through modelling

“Learning through modelling” captured the experience of students observing expert healthcare professionals partake in the simulation, who was setting a “standard of care” or displaying ideal practice. The behaviours, actions and interactions between team members demonstrated in the simulation form a “model” that students can then emulate in the workplace when they are qualified practitioners themselves. The subthemes within this main theme are “perceiving individual’s projected roles”, “interpersonal interactions”, “intrapersonal emotional regulation” and “patient-centredness”; e.g., “I learnt how a well-drilled team makes management of an emergency flow smoothly…” (MED 12, Table 2); “…the environment in an obstetric emergency can rapidly degenerate into one of panic…we need to be extremely proficient … so we can collect ourselves and respond adequately” (MED 39, Table 2).

### Dynamics of online interaction

This theme represents the interplay between medical and midwifery students and how the online setting of the workshop impacted both their communication with one another and on a deeper level, their perceptions of one another, including dominance in the discussion, the willingness to participate in interprofessional interactions, and mutual respect and appreciation in the workplace. The subthemes within this main theme are “dominance of a professional group”, “power imbalance relating to their greater knowledge and clinical experience than the medical student”, “equality of opinions”, “contribution from more clinically experienced midwifery students leading to increased learning by medical fraternity” and “awareness of clinical challenges” (Table 3).

## Discussion

The study evaluated student learning through online simulation workshops conducted over a 16-month period and found that the key learning outcomes between medical and midwifery students were similar to studies evaluating in-person simulation programs. The main themes arising from the evaluation of the online ONE-Sim workshops overlapped between medical and midwifery student groups but appeared to have a slightly different focus. Midwifery students indicated that they were more interested in interprofessional interactions, fostering positive relationships and a power balance, with the predominant theme being “dynamics of online interaction”. In contrast, medical students were most concerned with “learning through modelling” and “experiential learning”.

Social distancing measures implemented during the COVID-19 pandemic necessitated a transition to online learning. The online workshop format was designed on the concept of “learning through observation”, which has previously been studied.^14^ The majority of studies demonstrate that learning through observation is equally useful, or in some cases, better than “hands-on” participation in simulation, and is associated with high levels of learner satisfaction. Factors correlated with greater learning and satisfaction in the learning through observation framework include the use of observer tools, learner engagement and active participation in debriefing.^14^ We ensured these factors were considered when transitioning the ONE-Sim workshop from an in-person to an online format. An example of an observer tool is providing observers with an instructional briefing.^14^ In our study, this tool was implemented in the form of the “prebrief” (Figure 1) where participants were provided clear instructions on how the simulation would be conducted and particular elements of the simulation to pay attention to. When designing the structure of the online workshop, we ensured that a large proportion of the workshop was devoted to debriefing, an important element of successful "learning through observation” initiatives. Moreover, the majority of discussion within the debrief session was student driven to facilitate participants actively contributing to the debrief.

One of the greatest challenges in transitioning the ONE-Sim workshop to an online format was the familiarisation of facilitators and administrative with technologies such as video recording and video conferencing.  Though these technologies have existed for many years, they had not been used as extensively or regularly by educators prior to the pandemic. Specific challenges included ensuring accessibility to the link to the online workshop, camera angles enabled a clear view of the simulation, and facilitators were able to quickly and effectively launch breakout rooms in the debrief. Multiple practices of the simulation and review of the content by study investigators and administrative staff were required prior to the formal commencement of the program. It was observed that most medical students were more task-oriented, whereas midwifery students were more relationship-oriented. This could be due to inherent differences in learning styles. According to Kolb’s learning theory, there are four main learning styles: accommodating, diverging, assimilating and converging.^15^ Medical students have been shown to most commonly adopt the “assimilator” learning style.^16^ which is described as a logical approach to learning, where “ideas and concepts are more important than people”.^15^ By contrast, midwifery students mostly demonstrate a “diverging” learning style.^17^ described as “sensitive” and more focused on people.^15^ The predominant learning styles described amongst medical and midwifery students closely reflect the major themes arising in each disciplinary group.

The different learning outcomes from a shared educational experience demonstrated in this study are in keeping with an earlier paper reporting on an in-person IPE program similar in content and structure to the ONE-Sim program.^3^ The in-person workshop with medical and midwifery students demonstrated “learning by doing” as the key theme in medical students, while the midwifery students focussed around “power” and “roles and relationships”. This is similar to our current study, where key themes from medical students were related to the learning experience and through role modelling while midwifery students were focused on interaction with the medical students. This suggests the remote format of the obstetric interprofessional workshop may not significantly affect the main learning outcomes and patterns of focus between professional groups. Furthermore, it highlights both the strength of the association between profession and key learning areas and the realism to the original in-person simulation able to be achieved through the online format.

The findings also raise the possible difference in the occupation culture that may influence learning patterns in professional groups. Although medical students were in their fourth year of a five-year course, they are still a few years away from being able to practise as independent clinicians till they complete their speciality training; hence, their keenness to gain more procedure-based experience in early clinical learning years. By contrast, midwifery students are further into their clinical practice and have more experience in clinical birth settings. Hence, their focus is on how the interaction between colleagues can affect their future clinical practice. Midwifery students were particularly interested in the dynamics of online interactions. Due to their greater exposure to clinical practice, and discussion surrounding the interprofessional model of care early on in their training, some midwifery students may have drawn upon their own experiences in the workplace and were more aware of the challenges afflicting the doctor-midwife relationship. These were largely pertaining to mutual respect and appreciation for each profession’s skills and knowledge.

Acknowledgement of these challenges in a virtual setting provides evidence that e-learning may influence team-based attitudes and highlights the realism that can be created. Moreover, as was described, based on an in-person simulation program,^3^ midwifery students use their relative greater clinical and specialized theoretical knowledge and experience compared to medical students to gain respect for their profession and assert their power in the group. While medical and midwifery colleagues work closely together in a birth setting, their philosophy of learning may vary^18^ and possibly may drive divergent work culture.^19^ IPE at a student level may perhaps assist in closing this gap.

Both medical and midwifery students also expressed how the experiential learning aspect of the ONE-Sim program differed between the online and in-person formats. Unsurprisingly, both groups felt the online simulation of emergencies was not able to induce the same personal stress response as an in-person workshop potentially could have. This may be because the in-person ONE-Sim required students to perform the scenario themselves, compelling them to make critical decisions, communicate with the multidisciplinary team and carry out tasks under time pressurei20. By contrast, in the online ONE-Sim, students observe rather than partake in actively simulated emergencies and intercede as required, thus taking away the real-time experience and removing the anxiety of making ‘life-threatening’ decisions under time constraints. In effect, by managing the emergency themselves in the in-person workshop, students may have felt more accountable, increasing their own personal stress levels. Regulation of stress levels and greater ability to engage in rapid, accurate decision-making in obstetric emergency settings has been shown to improve neonatal and maternal outcomes.^21^ Reduced ability to gain experience in these important skills decreases the utility of online simulation programs when specifically addressing knowledge and experiential themes.

Although certain aspects of experiential learning may have been reduced through the online ONE-Sim, both groups, particularly medical students, noted online learning facilitated “learning through modelling”. Learning through modelling is becoming an increasingly recognized framework in healthcare education.^22^ The concept of a “role model” is founded upon two key theories. First, individuals are able to identify others holding a societal role or position to which they aspire to hold themselves.^22^ For the study participants, the senior obstetricians and midwives involved in the simulation, represented the roles that medical and midwifery students may have wished to hold themselves in their future practice. Secondly, the theory of social learning, where individuals actively observe role models’ behaviours as they believe this will assist them in learning advantageous skills and social norms in a particular context and adopt these behaviours themselves.^22^ By observing their chosen role model (obstetrician, neonatologist or midwife) in the simulated online emergencies, students developed greater understanding of the behaviours and demeanours that are expected of them in a professional environment, enabling them to implement these in their own future clinical practice.

Students commented not only on the individual behaviours of their role models but also on the interpersonal interactions occurring on a team level. The team simulating the emergency as a cohesive unit can also be considered a “role model” as students strive to become a member of the future multidisciplinary team. Thus, the communication, teamwork, role delegation and leadership demonstrated in the online workshop sets a standard of care and ideal practice that the students may emulate once they enter the workforce and become a member of an interprofessional team themselves. However, learning through role modelling in a simulation also poses the risk of students adopting poor or non-evidence-based practices that they may observe. Hence, it is important that students get an opportunity for open discussion as part of the learning process to allow different ideas to be heard, so they can identify and emulate best practice.  Both groups of students noted the broader applications of online learning. These include greater convenience and reduced travel time compared to in-person teaching sessions, consolidation of previous in-person learning; and improving consistency of clinical exposure. These benefits of online learning have implications for medical education practice beyond the pandemic. Implementation of online SBE and IPE programs may be considered in geographically remote areas where participation in face-to-face teaching may be limited by distance. Moreover, in remote and regional areas, there may be reduced availability of appropriately trained staff to facilitate such programs. The use of online SBE and IPE programs enables centralization of expert facilitators to deliver education to students in various geographical locations.

The results of our study support findings from the existing literature exploring the effectiveness of online learning models amongst healthcare students. As in previous studies,^23^ participants in our study found online learning a useful means of consolidating and enhancing their clinical skills and knowledge, captured in the theme “preparedness for practice”. In our study, students particularly felt that the debrief session held after the simulation, which enabled students to interact with one another and share ideas, was a useful means of improving communication skills and enhancing teamwork. These findings of improved communication and teamwork skills echo the results of an online program run at Harvard Medical School involving virtual “teams” that aimed to help the community and healthcare system during the pandemic.^24^ Furthermore, the theme of adaptability that arose in our study, particularly pertaining to the convenience and improved accessibility that online learning affords, has been reported in numerous studies.^23^ In light of these findings and the existing literature, online SBE and IPE programs may be considered for formal integration in healthcare education to supplement and enhance in-person teaching. Whilst there is a growing body of evidence demonstrating the effectiveness and advantages of online learning, it is important to note the potential adverse effects of online learning on mental illness amongst healthcare students. For example, a recent systematic review demonstrated that whilst the prevalence of anxiety amongst medical students during the pandemic remains similar to pre-pandemic, there are a number of stressors specific to the pandemic and online learning that correlate with higher anxiety levels.^25^ Online learning has been associated with fear of confinement and impaired ability to progress their education amongst students, contributing to increased levels of anxiety.^26^ Considering and mitigating these potential adverse effects is essential prior to the formal incorporation of online learning into the curriculum.

Limitations of our study include varying levels of clinical exposure of each group of workshop attendees at different stages of the pandemic. For example, students with greater clinical exposure may find the online ONE-Sim less helpful as they may have already developed or become familiar with the skills demonstrated in the workshop. A further limitation is the relatively low response rate amongst workshop attendees and the optional completion of the survey. Moreover, the majority of study participants had only attended the online ONE-Sim workshop. Therefore, it is difficult to directly compare students’ perceptions and learning from the online format to the in-person workshop. Finally, as our study uses a self-reported survey as the primary means of evaluation of the online workshop, there is a possibility that the findings are limited by both self-assessment and social desirability bias. For example, due to self-assessment bias, students may overestimate their perceived improvement in clinical skills or increased preparedness for practice, thereby overestimating the utility of and learning derived from online simulation workshops. Social desirability bias may particularly limit the findings of the reported experience of online interprofessional interactions through the workshop. For example, a medical student may report only the positives of learning alongside midwifery students without outlining the drawbacks of the interprofessional education, as this may be perceived as a more socially “desirable” or “acceptable” opinion. However, we hope to have minimized the effect of social desirability bias by using an anonymous survey.

## Conclusions

E-learning may be a valuable and meaningful way of delivering simulation-based interprofessional education. The study demonstrated that students were still able to achieve many of the learning objectives through the workshop, albeit through alternative learning methods, such as learning through modelling. While the interprofessional learning aspects of the workshop did not appear to suffer as a result of the online delivery format, the learning of actual hands-on procedural skills could not be adequately replaced in an online format.

The findings of our study have multiple implications in the evolving field of medical education. COVID is likely to persist for the foreseeable future, and as a result, there is an ongoing need for remote education alternatives in line with social distancing. Our study demonstrates online SBE and IPE programs are an effective and useful method of improving students’ preparedness for practice, delivering clinical skills education, and developing positive working relationships between professional groups during the pandemic. Beyond the pandemic, online SBE and IPE may be used in geographically remote areas where students may otherwise be required to commute long distances to receive SBE and IPE, or where there may be staffing issues that prevent easy access to SBE and IPE. Finally, online programs incorporating SBE and IPE may be used as an adjunct to in-person programs to enhance student learning and their overall learning experience. To better understand the impact of the online format on student learning, mixed methods study with a comparison to data based on in-person programs should be considered.

### Conflict of Interest

The authors declare that they have no conflict of interest.

## Supplementary material

Supplementary file 1 Appendix. The post-workshop survey
