# Supplementary material for: Evaluation of online interprofessional simulation workshops for obstetric and neonatal emergencies
Source: Int J Med Educ. 2022 Oct 31;13:287–304. doi: 10.5116/ijme.6342.9214 (PMC9911278; doi:10.5116/ijme.6342.9214)
Supplement: Supplementary file 1 — Appendix. The post-workshop survey [file ijme-13-287-S1.pdf]

Appendix

The post-workshop survey

ONE-Sim Post Workshop Student Survey

1. Age:
2. Gender:
3. Training (Medical or Midwifery)?
4. Please describe what you have learnt from this workshop and share your opinions on learning about obstetric emergencies via Zoom video conferencing rather than in person?
5. What do you think about gaining experience in interprofessional interactions via Zoom video conferencing?
6. What are your thoughts on a simulation workshop run via Zoom video conferencing that involves students only from your chosen profession (i.e., only midwifery students or only medical students)?
7. How do you think engaging with this workshop via Zoom video conferencing will impact the approach you take to your own clinical practice in future years?

Thank you for taking the time to fill out our survey!
